# Muscle oxygenation profiles between active and inactive muscles with nitrate supplementation under hypoxic exercise

**DOI:** 10.14814/phy2.13475

**Published:** 2017-10-25

**Authors:** Masahiro Horiuchi, Junko Endo, Shohei Dobashi, Yoko Handa, Masataka Kiuchi, Katsuhiro Koyama

**Affiliations:** ^1^ Division of Human Environmental Science Mt. Fuji Research Institute Fuji‐yoshida Japan; ^2^ Graduate School Department of Interdisciplinary University of Yamanashi Kofu Japan

**Keywords:** Blood flow, muscle O_2_ extraction, sympathetic vasoconstriction, tissue oxygenation

## Abstract

Whether dietary nitrate supplementation improves exercise performance or not is still controversial. While redistribution of sufficient oxygen from inactive to active muscles is essential for optimal exercise performance, no study investigated the effects of nitrate supplementation on muscle oxygenation profiles between active and inactive muscles. Nine healthy males performed 25 min of submaximal (heart rate ~140 bpm; EX_sub_) and incremental cycling (EX_max_) until exhaustion under three conditions: (A) normoxia without drink; (B) hypoxia (FiO_2 _= 13.95%) with placebo (PL); and (c) hypoxia with beetroot juice (BR). PL and BR were provided for 4 days. Oxygenated and deoxygenated hemoglobin (HbO_2_ and HHb) were measured in vastus lateralis (active) and biceps brachii (inactive) muscles, and the oxygen saturation of skeletal muscle (StO_2_; HbO_2_/total Hb) were calculated. During EX_sub_, BR suppressed the HHb increases in active muscles during the last 5 min of exercise. During EX_max_, time to exhaustion with BR (513 ± 24 sec) was significantly longer than with PL (490 ± 39 sec, *P *<* *0.05). In active muscles, BR suppressed the HHb increases at moderate work rates during EX_max_ compared to PL (*P *<* *0.05). In addition, BR supplementation was associated with greater reductions in HbO_2_ and StO_2_ at higher work rates in inactive muscles during EX_max_. Collectively, these findings indicate that short‐term dietary nitrate supplementation improved hypoxic exercise tolerance, perhaps, due to suppressed increases in HHb in active muscles at moderate work rates. Moreover, nitrate supplementation caused greater reductions in oxygenation in inactive muscle at higher work rates during hypoxic exercise.

## Introduction

It has been suggested that nitric oxide (NO) is a major factor in hypoxic‐induced vasodilation (Casey et al. 2010). Accumulating evidences revealed that dietary inorganic nitrate (NO_3_^−^) has been linked to many physiological benefits, including improvement of oxygen supply to peripheral tissue (Hord et al. 2009). It is also widely accepted that NO_3_^−^ is reduced to nitrite (NO_2_^−^) and further to NO, generally known as NO_3_^−^‐NO_2_^−^‐NO pathway (Lundberg and Weitzberg 2010). Given these biological effects, recent studies have investigated whether dietary nitrate (NO_3_^−^) supplementation (e.g., beetroot juice rich with NO_3_^−^), which is a potent vasodilator, improves exercise performance or has other ergogenic effects under hypoxia. Some studies showed that that dietary NO_3_^−^ supplementation reduced oxygen cost and/or improved exercise tolerance under hypoxia (Vanhatalo et al. 2011, 2014; Masschelein et al. 2012; Kelly et al. 2014; Muggeridge et al. 2014; Shannon et al. 2016, 2017); others, however, had contradictory findings (Arnold et al. 2015; Bourdillon et al. 2015; MacLeod et al. 2015; Carriker et al. 2016; Nyback et al. 2017). One possible explanation to account for these discrepancies may relate to differential study settings. For example, previous studies (e.g., Kelly et al. 2014) used various range of oxygen levels, fraction of inspiratory oxygen (FiO_2_) = 0.11–0.16, and it should be noted that subjects in most of previous studies were exposed to hypoxic condition for a relative short duration, ~15 min before the main exercise test except one study (Masschelein et al. 2012).

At high‐altitude, initial compensatory physiological responses is an increase in pulmonary ventilation to deliver sufficient oxygen into peripheral tissues (Dempsey and Foster 1982) and this immediate increases in ventilation showed a stable about after 1 h hypoxic exposure (Easton et al. 1986). These may indicate that a short duration of resting baseline in most of previous studies (e.g., Kelly et al. 2014; ~10 min hypoxic exposure) may cause different resting condition between subjects, resulted in controversial findings. In this regard, Masschelein et al. (2012) took 1 h resting hypoxic exposure before exercise, and found that dietary nitrate supplementation improves muscle oxygenation status, but not cerebral oxygenation status during hypoxic exercise. As it is well known that blood flow redistribution from inactive to active muscles is essential to optimize exercise performance (Rowell 1993), it may be required to investigate interaction in oxygenation status between these muscles for further understanding to elucidate potential mechanisms of nitrate effects on exercise performance in hypoxia.

In resting muscles during exercise, sympathetic vasoconstriction is known to be well preserved under either normoxia or hypoxia (Remensnyder et al. 1962; Hansen et al. 2000), a phenomenon for which Remensnyder et al. (1962) coined the term functional sympatholysis. Exercise training‐induced improvement of functional sympatholysis increases the maximal oxygen uptake of animal models (Mizuno et al. 2014). These results suggest that the balance between metabolic vasodilation in active muscles and sympathetic vasoconstriction in inactive muscles may play an important role in optimizing exercise performance. Thus, enhanced sympathetic vasoconstriction in inactive muscles may support oxygen supply via vasodilation in active muscles, and thus improve performance in hypoxia. Conversely, if nitrate supplementation may cause a vasodilation in inactive muscles as well as in active muscles, this may counteract improvement of exercise performance in hypoxia.

The aim of this study was to examine the potential impact of dietary nitrate supplementation on hypoxic exercise performance, as well as the interaction between metabolic vasodilation in active and sympathetic vasoconstriction in inactive muscles during submaximal and maximal incremental exercise in healthy young humans. We used near‐infrared spectroscopy (NIRS) to evaluate tissue oxygenation with high temporal resolution, since changes in tissue oxygenation have been shown to be a reliable measure of sympathetic vasoconstriction in resting and exercising skeletal muscles (Hansen et al. 1996; Fadel et al. 2004; Ogata et al. 2007, 2008; Horiuchi et al. 2014, 2015). In addition, muscle deoxygenation has been suggested to be an indicator of muscle O_2_ extraction (*a‐v* O_2_ difference) (DeLorey et al. 2003; Grassi et al. 2003). We evaluated muscle O_2_ extraction at active muscles and sympathetic vasoconstriction at inactive muscles using NIRS. We hypothesized that dietary NO_3_^−^ supplementation would improve exercise performance under hypoxia via lower exercise‐induced increases in muscle deoxygenation in active muscles (Masschelein et al. 2012) despite higher muscle oxygenation levels in inactive muscles.

## Methods and Material

### Subjects

Nine healthy male subjects with a mean age of 21 ± 3 years, height of 176 ± 5 cm, and body mass of 73 ± 9 kg (mean ± SD) participated in this study. Subjects engaged in regular physical activity (1–2 h per day, 3–5 days per week). None of the subjects had been exposed to an altitude higher than 1500 m within 6 months prior to the study. After receiving a detailed description of all study procedures and the possible risks and benefits of participation, each subject signed an informed consent form. All procedures were approved by the ethical committee of Mt. Fuji Research Institute in Japan and were performed in accordance with the guidelines of the Declaration of Helsinki (ECMFRI‐01‐2014).

### Experimental procedures

Subjects were requested to abstain from caffeinated beverages for 12 h, and from strenuous exercise and alcohol for a minimum of 24 h before each session. All studies were performed at a temperature of 24 ± 1°C, and external stimuli were minimized. All subjects performed three trials: (1) normobaric normoxic exercise without any drink (Norm); (2) normobaric hypoxic exercise (FiO_2_ = 0.1395) with a placebo drink (PL), and (3) normobaric hypoxic exercise (FiO_2 _= 0.1395) with beetroot juice (BR). Each subject performed the Norm trial first. The two hypoxic trials were performed afterward in a random order. All three trials were performed with at least a 2‐week washout period. For 3 days prior to the BR and PL trials, subjects consumed 140 mL/day of NO_3_^−^‐rich BR or 140 mL/day of NO_3_^−^‐depleted BR concentrate as a placebo drink (Beet It; James White Drinks, Ltd., Ipswich, UK) (Kelly et al. 2014). Both subjects and researchers were blinded to the drink contents until the completion of the study. Subjects were also provided with a list of foods rich in NO_3_^−^ and instructed to avoid the consumption of these foods and to maintain their normal dietary intake for the duration of the study. In addition, they were asked to abstain from the use of antibacterial mouthwash, which eliminates the oral bacteria that reduce NO_3_^−^ to NO_2_^−^ for the duration of the study (Govoni et al. 2008).

On each study day (PL or BR trails), subjects consumed their final dose of BR or PL upon arrival at the laboratory, 2.5 h prior to the start of their submaximal exercise. Venous blood was sampled 30 min after the final dose (see *“Blood samples and analysis”* section below). This ingestion time was previously shown to achieve a peak increase in plasma nitrates or nitrite concentration that improved exercise tolerance (Wylie et al. 2013).

**Figure 1:**
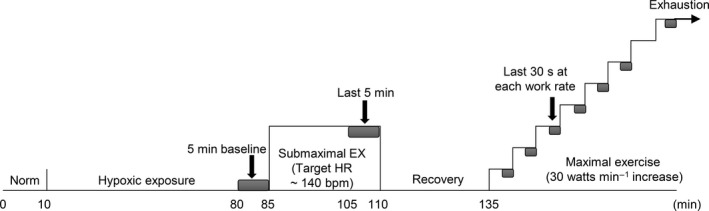
Experimental protocol of hypoxic trails (placebo and beetroot) in this study. Norm, normoxia; EX, exercise; HR, heart rate; bpm, beats per minute. Gray squares indicate the values at baseline, the last 5 min during submaximal exercise, and the last 30 sec at each work rate during maximal exercise. Data for the last 5 min and last 30 sec were used to calculate relative changes from the baseline values in near infrared spectroscopy signals (see Data analysis in the text).

In all three trials (Norm, PL, and BR), 10 min normoxic resting condition was set at first while breathing room air. During the PL and BR trials, after 10 min normoxic exposure hypoxic gas was supplied via a commercial tent (about 4000 L) in tandem with a hypoxic gas generator system (YHS‐B05S: YKS, Nara, Japan; and Hypoxico Everest Summit II: Will Co., Ltd., Tokyo, Japan). Hypoxic baseline values were measured over the last 10 min of a 75‐min resting hypoxic exposure. Inspired oxygen concentration was verified before and after each experiment (AE‐300; Minato Medical Science, Osaka, Japan). After baseline measurements were acquired, subjects performed submaximal exercise on a cycle ergometer (Ergomedic 828 E; Monark, Stockholm, Sweden) in a semi‐recumbent position for 25 min at a target heart rate (HR) of 140 bpm. The pedaling rate was set as 60 rpm. Briefly, the starting work rate was 30 W, and it was increased by 30 W every minute, up to 5 min, until the subject's HR reached the target of 140 bpm. After reaching this HR, the subjects continued to cycle at a constant pedaling rate of 60 rpm for 25 min and exercise intensity was manually adjusted to maintain the target HR while the 25‐min cycling (Ogoh et al. 2014; Komiyama et al. 2015). Subjects then rested for 25 min before performing a maximal incremental exercise test (the starting work rate was 30 W, and increased by 30 W/min) until exhaustion. Subjects were asked to maintain a pedal frequency of 60 rpm throughout the exercise. The criteria for exhaustion was were as follows: (1) No increase in VO_2_ despite a further increase in work rate; (2) HR at 90% of the age‐predicted maximal value (220‐participant's age); (3) A rating of 19 on the Borg's scale of perceived exertion; or (4) Failure to maintain pedaling frequency of 60 rpm despite strong verbal encouragement. When participants met at least two of the above criteria, the test was terminated (Dobashi et al. 2016). Study procedures are shown in Figure 1.

### Measurements

Pulmonary ventilation (*V*_E_) and gas exchange variables were measured with a breath‐by‐breath metabolic measurement system (AE‐310S; Minato Medical Science, Osaka, Japan), which assessed inspired and expired gas volumes via hot‐wire respirometry. Flow signals were electrically integrated for the duration of each breath, and summed to calculate minute ventilation. The expired fractions of O_2_ and CO_2_ were analyzed using a zirconium solid electrolyte oxygen analyzer and an infrared carbon dioxide analyzer, respectively. The standard gases (O_2_ 15.23%, CO_2_ 4.999%, and N_2_ balance) and room air were used to calibrate the gas analyzer.

Throughout the study, participants' HR were recorded with a wireless HR monitor (Polar RC800X; Polar Electro Japan, Tokyo, Japan). Arterial O_2_ saturation (SpO_2_) was monitored by a pulse oximeter on the right middle finger every 1 min throughout the study (Pulsox‐3; Minolta, Tokyo, Japan). Local tissue oxygenation profiles of the vastus lateralis (active) and biceps brachii (inactive) muscles were measured using NIRS (BOM‐L1TRW; Omegawave, Tokyo, Japan), as previously described (Horiuchi et al. 2014, 2015). In this study, each subject performed cycling in a semi‐recumbent position, and their arms were placed on side tables rather than on the handlebars. In addition, their arms were completely fixed by a vacuum pack to prevent arm movement (Vacuform, Muranaka Medical Instruments Co., Ltd., Osaka, Japan), and thus their biceps brachii muscles could be classified as inactive.

The NIRS instrument used three laser diodes (780, 810, and 830 nm) and calculated relative tissue levels of oxygenated and deoxygenated hemoglobin (HbO_2_ and HHb) according to the Modified‐Beer‐Lambert law. Total Hb was calculated as the sum of HbO_2_ and HHb, and the oxygen saturation of skeletal muscle (StO_2_) was expressed as (HbO_2_/total Hb) × 100 (i.e., as a percentage). NIRS optodes were placed over the lower third of the vastus lateralis muscle (10–12 cm above the knee joint) (Koga et al. 2007), and the belly of the biceps brachii muscle on the upper left arm of each subject (Ogata et al. 2007). The probe holder contained one light source probe, and two detectors were placed 2 cm (detector 1) and 4 cm (detector 2) away from the source. Hb concentrations received by detector 1 were subtracted from those received by detector 2. This procedure minimized the influence of skin blood flow (SkBF) (Ando et al. 2010). In this device, path wave length was set as 4.0; however, as modified beer lambert method cannot assess actual path wave length (Van der Zee et al. 1992), meaning that provisional absolute values, for example, *μ*mol/L, are estimated values, we showed the NIRS values as arbitrary unit in representative recordings (see in Fig.  3), and relative changes from baseline values in mean values (see in data analysis and Figs. 4, 5).

It has been reported that NIRS signals can reach half the depth of the distance between the probe and detector (Patterson et al. 1989). With this in mind, we used a distance of 4 cm between probes, which provided an NIRS signal traversing approximately 20 mm. This allowed the appropriate depth for sample muscles, since the sum of the skinfold plus the muscle thickness in the biceps brachii and vastus lateralis muscle was over 20 mm. Indeed, the measured skinfold thicknesses of the biceps brachii and vastus lateralis muscles were 3.8 ± 1.2 mm and 4.1 ± 1.0 mm, respectively (*P *=* *0.073), whereas the muscle thickness was 28.4 ± 3.8 mm for the biceps brachii and 30.7 ± 5.3 mm (mean ± SD) for the vastus lateralis (*P *=* *0.084), as assessed by B‐mode ultrasound (logic‐e; GE Healthcare, Tokyo, Japan). These numbers indicate that when NIRS probes were placed over the skin of each muscle, the NIR light was indeed transmitted to the desired muscle bed. Both muscles with attached optodes and covering were wrapped with an elastic bandage to minimize optode movement, while permitting mobility for cycling. SkBF at the vastus lateralis muscle was recorded using the laser Doppler method (ATBF‐LC1; Unique Medical Co., Ltd., Tokyo, Japan). The electrodes were briefly placed over the skin, about 2‐cm away from the NIRS probe. Pen marks were made on the skin to indicate the margins of the probe holder and electrodes. All subjects were asked to mark themselves again between trials before washing so that the optodes could be positioned at exactly the same place for each test.

Blood pressure (BP) was measured using two different methods. At rest, BP was measured with the oscillometric method using a digital BP monitor (HEM‐907; Omron, Tokyo, Japan) on the upper portion of each patient's right arm. BP was measured at least three times with a 1‐min interval between measurements. If the difference between the measurements of either systolic or diastolic BP was >5 mmHg, the measurements were repeated. The average BP values of measurement pairs were taken as the BP values, excluding those that were >5 mmHg. Under the PL and BR conditions, BP was measured under both normoxia and hypoxia during the resting periods. During exercise, beat‐by‐beat BP was also measured using finger photoplethysmography at the middle or index finger of the left hand (MUB‐101; Medisens Inc., Tokyo, Japan) throughout the study.

Data on the beat‐by‐beat BP, NIRS signals, and SkBF were stored with a sampling frequency of 200 Hz by a field data recorder (es8; TEAC, Tokyo, Japan), and transferred to a laptop computer for further analysis.

### Blood samples and analysis

Resting venous blood samples (10 mL) were taken from the antecubital vein and immediately centrifuged at 1000 *g* for 15 min at 4°C (MX‐300; Tomy Seiko Co., Ltd., Tokyo, Japan) to separate serum and plasma. The serum samples were frozen at −80°C for further analysis of NO_3_^−^ by SRL Co., Ltd. (Tokyo, Japan). Capillary blood samples (0.3 *μ*L) were taken from the finger for blood lactate concentration (LA) measures. Using an automated lactate analyzer, LA was analyzed immediately at rest under normoxia (Norm) and hypoxia (PL and BR), after 5 min of submaximal exercise, and after 5 min of maximal leg cycling (Lactate Pro 2LT‐1730; Arkray, Tokyo, Japan).

### Data analysis

At rest, all physiological values (i.e., mean values for gas exchange variables, HR, SpO_2_, and the NIRS signals) were calculated over the last 5 min before the submaximal exercise in each trial (resting baseline values). Data were averaged over the last 5 min of submaximal exercise as the work rate was held to be constant during the last 5 min. During the maximal exercise, data were averaged over the last 30 sec at each work rate up to 180 W, and the last 30 sec just prior to exhaustion. To compare NIRS signals between subjects, the changes in HbO_2_, HHb, and total Hb were quantified as percentages from the resting baseline values. Because our NIRS device can represent each NIRS signal as an arbitrary unit, resting baseline values were defined as 100% and differences were shown as relative changes (Horiuchi et al. 2016) (Fig. 1). Using the oscillometric method, mean arterial pressure (MAP) was calculated as ([2 × diastolic pressure] + systolic pressure)/3, while beat‐by‐beat MAP was measured as the time averaged from the beat‐by‐beat pressure wave.

### Statistics

Data were presented as the means ± SD. Based on the study design, separate analyses using two‐tailed paired *t*‐tests were performed to compare the effects of dietary NO_3_^−^ supplementation (BR vs. PL) and the general effects of hypoxia (Norm vs. PL) for comparison of cardiorespiratory variables and NIRS signals during submaximal exercise (Masschelein et al. 2012). A two‐way repeated measures ANOVA was also used to compare exercise‐induced relative changes in NIRS signals during maximal exercise. A *P* < 0.05 was considered statistically significant.

## Results

### General effects of hypoxia (Norm vs. PL)

**Figure 2:**
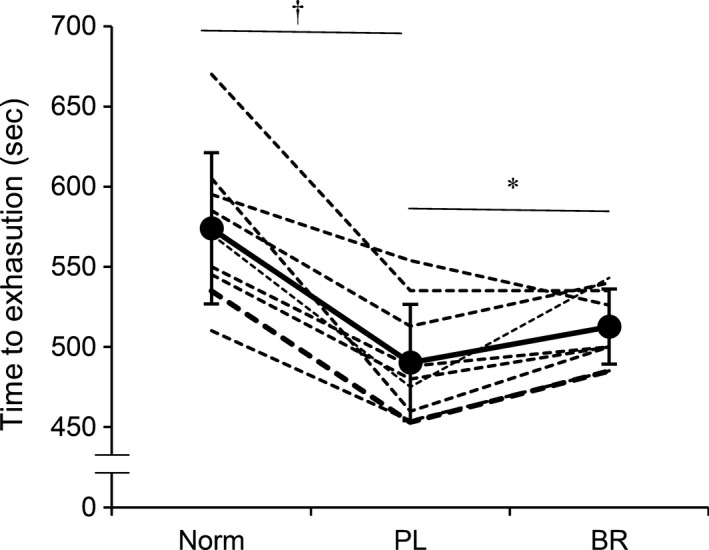
Time‐to‐exhaustion during maximal incremental exercise under normoxia (Norm), after placebo (PL) and beetroot (BR) supplementation. Dotted lines indicate an individual data, and the solid line indicates averaged values. Values are the means ± standard deviation (SD*)*. *P <* *0.05 between PL and BR, †*P *<* *0.05 between Norm and PL.

At rest, 75 min of hypoxia reduced resting SpO_2_ and increased MAP (*P *<* *0.05), while there were no differences in other variables (Table 1). During EX_sub_, the values of gas exchange variables and SpO_2_ were lower under the PL than the Norm condition (*P *<* *0.05). Similar results were also observed during EX_max_ (*P *<* *0.05, Table 1). In addition, hypoxic conditions significantly impaired exercise tolerance, time‐to‐exhaustion (574 ± 47 in Norm vs. 490 ± 39 sec in PL, *P *<* *0.001), and maximal work rate (287 ± 34 in Norm vs. 233 ± 25 W in PL, *P *=* *0.002) (Fig. 2).

**Table 1:** Cardiorespiratory and hemodynamic variables under normoxia, placebo and beetroot trials at rest and during submaximal and maximal exercise

	Normoxia	Placebo	Beetroot	*P* value	
				Norm vs. PL	PL vs. BR
Rest					
*V*O_2_, mL/min	297 ± 33	305 ± 47	296 ± 40	0.622	0.431
*V*CO_2_, mL/min	257 ± 30	277 ± 42	273 ± 38	0.257	0.788
RER	0.87 ± 0.07	0.92 ± 0.04	0.92 ± 0.06	0.207	0.807
*V*_E_, L/min	10.0 ± 1.4	10.8 ± 1.4	10.9 ± 1.7	0.183	0.919
HR, bpm	60 ± 7	64 ± 7	65 ± 5	0.058	0.757
MAP, mmHg	85 ± 5	88 ± 6^b^	86 ± 7	<0.001	0.085
LA, mmol/L	1.4 ± 0.3	1.6 ± 0.2	1.7 ± 0.2	0.206	0.122
SpO_2_, %	98.3 ± 0.8	88.1 ± 2.0^b^	87.6 ± 1.5	<0.001	0.590
Submaximal exercise					
*V*O_2_, mL/min	1908 ± 305	1442 ± 183^b^	1382 ± 225	<0.001	0.209
*V*CO_2_ mL/min	1744 ± 286	1384 ± 161^b^	1277 ± 207^a^	<0.001	0.012
RER	0.91 ± 0.02	0.97 ± 0.07	0.93 ± 0.07	0.063	0.069
V_E_, L/min	48.5 ± 8.9	46.3 ± 5.7	44.4 ± 10.0	0.371	0.344
HR, bpm	140 ± 2	140 ± 4	141 ± 4	0.715	0.560
MAP, mmHg	100 ± 10	93 ± 5	92 ± 8	0.080	0.843
SkBF, %	544 ± 223	538 ± 222	560 ± 321	0.953	0.744
LA, mmol/L	2.8 ± 1.1	4.0 ± 2.1^b^	3.8 ± 1.6	0.040	0.759
SpO_2_, %	96.6 ± 1.3	74.6 ± 5.2^b^	73.8 ± 5.1	< 0.001	0.592
Maximal exercise					
*V*O_2 peak_, mL/min	3127 ± 361	2450 ± 277^b^	2612 ± 337	<0.001	0.066
*V*CO_2 peak_, mL/min	3688 ± 457	3152 ± 416^b^	3222 ± 301	0.003	0.516
RER _peak_	1.18 ± 0.06	1.29 ± 0.17	1.23 ± 0.09	0.105	0.254
*V*_E__peak_, L/min	111.3 ± 20.1	117.5 ± 17.0	118.0 ± 16.6	0.210	0.914
HR _peak_, bpm	180 ± 9	178 ± 6	182 ± 5^a^	0.352	0.031
MAP, mmHg	149 ± 14	133 ± 20	130 ± 16	0.071	0.441
SkBF, %	529 ± 287	535 ± 287	568 ± 222	0.950	0.832
LA _peak_, mmol/L	12.1 ± 1.7	10.3 ± 2.6	12.3 ± 2.8	0.064	0.078
SpO_2 nadir_,%	95.3 ± 1.5	69.4 ± 5.9^b^	68.9 ± 6.1	<0.001	0.683

Values are the means ± standard deviation (SD).

Norm, normoxia; PL, placebo; BR, beetroot; *V*O_2_, oxygen uptake; *V*CO_2_, carbon dioxide output; RER, respiratory gas exchange ratio; *V*_E_, ventilation; HR, heart rate; MAP, mean arterial pressure; LA, blood lactate concentrations; SpO_2_, arterial O_2_ saturation SkBF, skin blood flow in active muscle. Note that MAP was measured by the oscillometric method at rest.

a *P *<* *0.05 between placebo and beetroot.

b *P *<* *0.05 between normoxia and placebo.

### Nitrate concentration and resting cardiorespiratory variables (PL vs. BR)

Nitrate supplementation (BR) significantly increased NO_3_^−^ concentration compared to PL (NO_3_^−^37.2 ± 23.9 with PL vs. 220.0 ± 79.5 mmol/L with BR, *P *<* *0.001), while BR had no observable effect on cardiorespiratory variables (all *P *>* *0.05, Table 1).

### Effects of nitrate supplementation during EX_sub_ (PL vs. BR)

**Figure 3:**
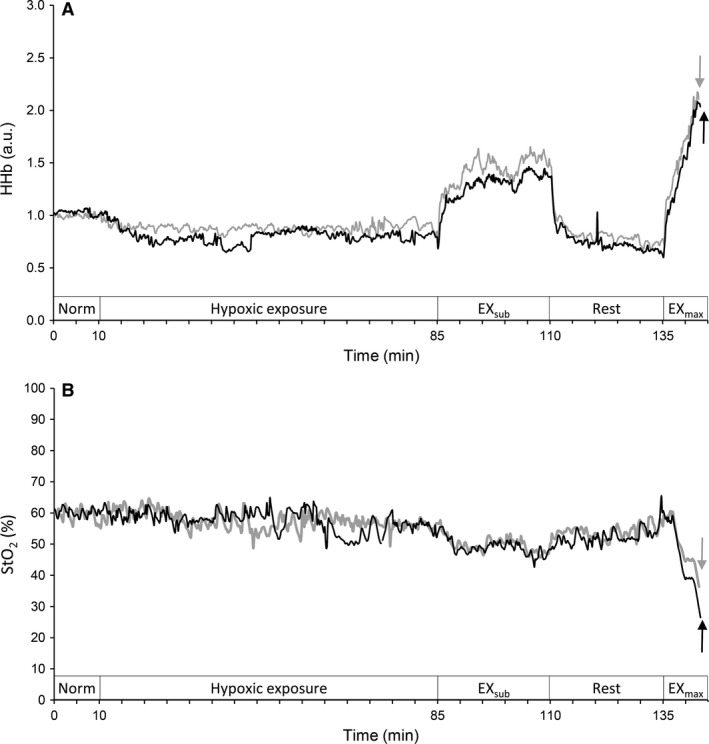
Time course changes in muscle deoxygenation (HHb) status in active muscles (vastus lateralis) and saturation of skeletal muscle (StO_2_) in inactive muscles (biceps brachii) for a typical single subject. Gray lines indicate PL and black lines indicate BR trials, respectively. a.u., arbitrary unit; EX_sub_, submaximal leg cycling exercise; EX_max_, incremental leg cycling exercise. Arrows indicate at the point of exhaustion.

**Figure 4:**
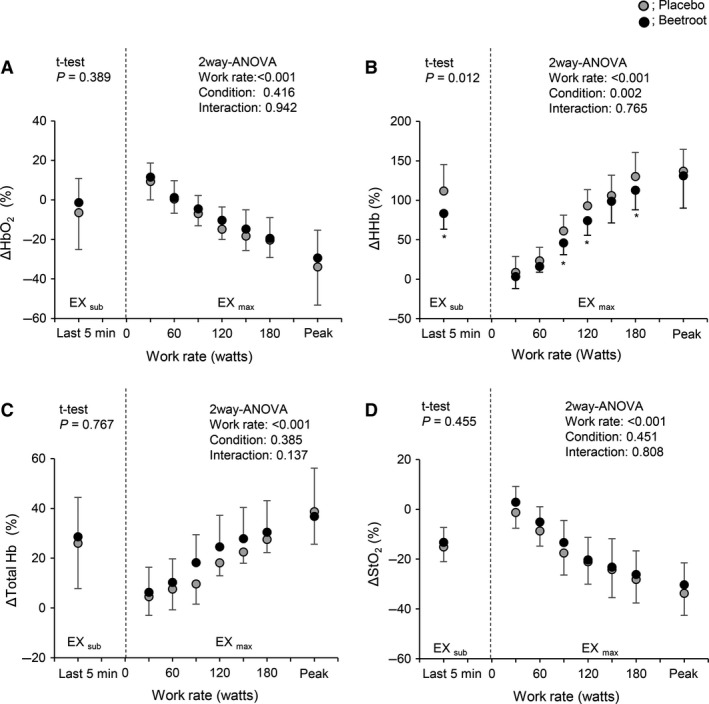
Relative changes from baseline values in each near infrared spectroscopy signal in active muscle during EX_sub_ and EX_max_ between PL and BR trials. HbO_2_, muscle oxygenation; Total Hb, total hemoglobin. Peak indicates the individual peak work rate. Values are the means ± SD. *P <* *0.05 between PL and BR within the same work rates.

**Figure 5:**
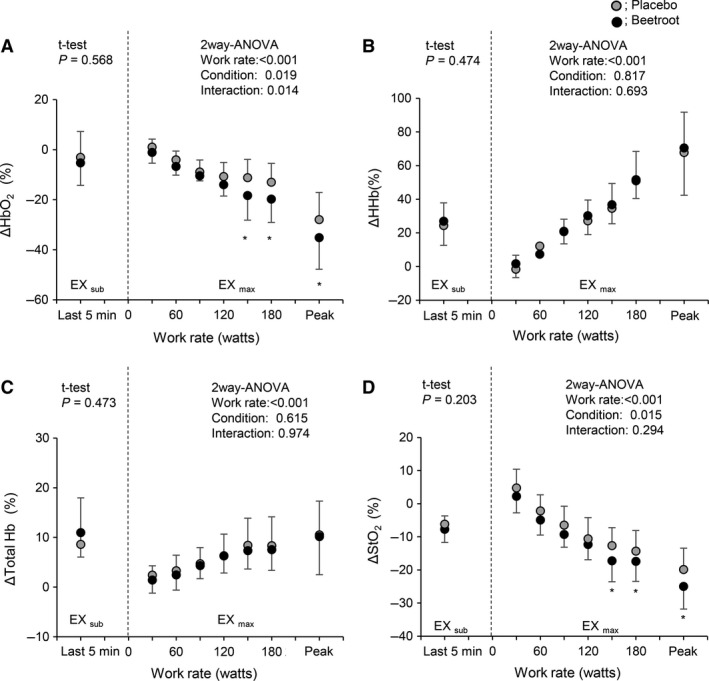
Relative changes from baseline values in each near infrared spectroscopy signal in inactive muscle during EX_sub_ and EX_max_ between PL and BR trials. Values are the means ± SD. * *P *<* *0.05 between PL and BR within the same work rates.

During EX_sub_, the work rate in the last 5 min was similar between PL and BR (105 ± 13 under PL vs. 105 ± 17 W under BR, *P *=* *0.924). *V*CO_2_ with BR was significantly lower during EX_sub_ compared to PL (*P *=* *0.016, Table 1), while the other cardiorespiratory variables were unchanged during EX_sub_ between BR and PL (all *P *>* *0.05). Time course changes in HHb in active muscles (Fig. 3A) and StO_2_ in inactive muscles (Fig. 3B) for a typical single subject are shown. During EX_sub_, HHb in active muscles with BR appeared to be lower, while, StO_2_ in inactive muscles showed similar values. Figure 4 shows mean values of relative changes in each NIRS signal in active muscle during the last 5 min of EX_sub_. BR supplementation suppressed delta increases in HHb from the baseline during the last 5 min of EX_sub_ (111.7 ± 33.7% with PL vs. 83.3 ± 19.9% with BR, *P *=* *0.012). In contrast, there were no significant differences in the changes of HbO_2_, total Hb, and StO_2_ in active muscle between PL and BR (all *P *>* *0.05). Mean values of the relative changes in each NIRS signal in inactive muscle during the last 5 min of EX_sub_ are shown in Figure 5. There were no differences in all NIRS metrics between PL and BR during EX_sub_ (all *P *>* *0.05).

### Effects of nitrate supplementation during EX_max_

Nitrate supplementation (BR) significantly improved the time‐to‐exhaustion compared to PL (513 ± 24 in BR vs. 490 ± 39 sec in PL, *P *=* *0.036, Fig. 2); no differences were observed in maximal work rate between BR (257 ± 22 W) and PL (233 ± 25 W, *P *=* *0.133).

HR_peak_ was significantly higher (*P *=* *0.031) and *V*O_2peak_ was slightly but not significantly higher (*P *=* *0.066) under the BR versus the PL condition during EX_max_ (Table 1). There were no statistically significant differences in other variables between PL and BR during EX_max_ (all *P *>* *0.05, Table 1).

HHb in active muscles during EX_max_ seems to be slightly lower with BR (Fig. 3A) and StO_2_ in inactive muscles decreased greater with BR compared to PL (Fig. 3B). Mean values of the relative changes in each NIRS signal in active (Fig. 4) and inactive (Fig. 5) muscle during the last 30 sec at each work rate of EX_max_ are shown. In active muscle, HHb showed significantly lower values at the work rates of 90, 120 and 180 W with BR supplementation (~ 18% lower with BR, *P *<* *0.05). In contrast, there were no significant differences in the changes of HbO_2_, total Hb, and StO_2_ in active muscle between PL and BR during EX_max_ (all *P *>* *0.05). In inactive muscles, relative decreases in HbO_2_ and StO_2_ were significantly greater by ~7% in HbO_2_, and ~5% in StO_2_ with BR supplementation compared to PL at higher work rates (>150 W) with statistical differences between conditions (*P *<* *0.05). No differences were observed in HHb and total Hb between PL and BR (all *P *>* *0.05).

## Discussion

This study is the first to investigate the effects of dietary nitrate supplementation on muscle oxygenation profiles between active and inactive muscles during hypoxic exercise. Our results indicate that, compared to PL, BR supplementation improved exercise tolerance, and reduced HHb in active muscles at moderate work rates. Moreover, reductions in tissue oxygenation in inactive muscles were greater during EX_max_ with BR supplementation than with PL.

### Effects of nitrate supplementation during EX_sub_ (PL vs. BR)

According to the Fick equation, oxygen uptake can be determined as a function of O_2_ delivery and O_2_ extraction, and HHb has been suggested to be an indicator of muscle O_2_ extraction (*a‐v* O_2_ difference) (DeLorey et al. 2003; Grassi et al. 2003). In this study, because HR was set as constant value of around 140 bpm during exercise, lower values in HHb in active muscles and/or lower stroke volume with BR may have produced lower *V*O_2_. However, a recent study demonstrated that BR supplementation did not change stroke volume during exercise (Hirai et al. 2017) even though different study settings between their study and our study, that is, heart failure patients versus healthy active subjects, and normoxia versus hypoxia. Thus, it may be still possible that lower *V*O_2_ values ~5% with BR compared to PL may relate to lower HHb. Indeed, similar low *V*O_2_ values of ~5%, were also observed in previous studies during hypoxic exercise (Masschelein et al. 2012; Kelly et al. 2014; Muggeridge et al. 2014) and lower HHb was also observed (Masschelein et al. 2012). These results may indicate that sufficient O_2_ delivery with nitrate supplementation, resulted in a lower rate of muscle O_2_ extraction. We also found lower VCO_2_ with BR supplementation. As it has been reported that increases in VCO_2_ accounted for the anaerobically‐generated excess‐CO_2_ (Kisaka et al. 2015), lower VCO_2_ might indicate no increases in anaerobic glycolysis to energy turnover with BR supplementation.

### Effects of nitrate supplementation during EX_max_ (PL vs. BR)

In agreement with previous studies, we found that dietary nitrate supplementation significantly improved hypoxic exercise tolerance (Masschelein et al. 2012; Kelly et al. 2014). Other previous studies also demonstrated that nitrate supplementation significantly improved exercise tolerance under normoxia (Bailey et al. 2009, 2010; Lansley et al. 2011; Breese et al. 2013). In this study, BR supplementation suppressed the increases in HHb at moderate work rates compared to PL. Although we can only speculate, these results also suggested that muscle O_2_ extraction was restrained likewise during EX_sub_, perhaps, due to enhanced blood flow to active muscles with nitrate supplementation. In partly support this, total Hb was slightly higher with BR compared to PL though no statistical differences were observed. Because total Hb which was calculated by the sum of HbO_2_ and HHb, that is, blood volume, was correlated with changes in tissue blood flow (Van Beekvelt et al. 2001). While some authors have reported that a rightward shift (Bohr effect) in the oxyhemoglobin dissociation curve accelerates the rate of deoxygenation at or near lactate or ventilatory thresholds (Belardinelli et al. 1995; Grassi et al. 1999). The results of lower HHb at moderate work rate during EX_max_ appeared to have more muscle O_2_ extraction reserve, which resulted in the extension of time‐to‐exhaustion.

Contrary to our hypothesis, BR supplementation caused greater reductions in StO_2_ (*P *=* *0.001) and HbO_2_ (*P *=* *0.040) at exhaustion in inactive muscles compared to PL. Tissue oxygenation reflects the local balance between O_2_ supply and O_2_ consumption, and thus the decreased HbO_2_ and StO_2_ in inactive muscles may reflect a reduction in O_2_ supply to the inactive muscles under the assumption that O_2_ consumption would be constant in resting muscles (Ogata et al. 2002). Moreover, our results may indicate that sympathetic vasoconstriction was augmented by BR supplementation, as reductions in tissue oxygenation have been shown to be an indicator of sympathetic vasoconstriction with acute sympathetic stimulation during small muscle exercise (Hansen et al. 1996; Fadel et al. 2004; Horiuchi et al. 2014, 2015). With regard to whole body exercise, one also found that tissue oxygenation decreased at higher intensity exercise, for example, above anaerobic threshold, during incremental leg cycling (Ogata et al. 2007). Although direct and enough evidence may be required if reductions in tissue oxygenation in inactive muscles during whole body exercise is related to sympathetic vasoconstriction, Sheel et al. (2002) reported that inactive leg blood flow at rest was reduced when the fatiguing inspiratory muscle work was loaded. They suggested that the sympathetic vasoconstriction can be accounted for the decrease in the inactive leg blood flow during the inspiratory work because St Croix et al. (2000) found that the sympathetic nerve activity (SNA) was increased by the same inspiratory muscle work. It has also been reported that fatigue of the diaphragm occurs during whole body endurance exercise in excess of 85% of maximal oxygen uptake (Johnson et al. 1993). Together, the results in this study which showed greater reductions in HbO_2_ and StO_2_ in inactive muscles at higher exercise intensity with BR may relate to enhanced SNA‐induced vasoconstriction. Although we have no direct evidence to explain this, our recent study showed that ischemic preconditioning (IPC) caused a greater reduction in muscle oxygenation in resting muscles with acute sympathetic stimulation (cold pressor test). Moreover, the subjects with greater reduction in oxygenation in resting muscles showed greater increases in oxygenation in exercising muscles, suggesting that sympathetic vasoconstriction in resting muscles may have an important role to optimize exercise performance (Horiuchi et al. 2015). In addition, this IPC protocol could prevent exercise‐induced decrease in endothelial function, probably, due to enhanced nitric oxide bioavailability (Bailey et al. 2012). When relating such findings to our observations, benefits of experimental manipulation for increased NO bioavailability, that is, nitrate supplementation, might improve exercise performance via greater reductions in oxygenation in resting muscles. Indeed, greater reductions in oxygenation in even small inactive muscles (i.e., arm muscles) may cause higher peak VO_2_ during leg cycling (Yano et al. 2005), and a given increased in sympathetic nerve activity in inactive muscles may be required for optimal blood flow redistribution to active muscles for maintenance during knee extension exercise (Keller et al. 2003, 2004).

In addition, if increases in HR broadly reflect leg blood flow during cycling, as has been suggested for a knee‐extension exercise (MacPhee et al. 2005), higher HR with BR in this study may relate to effective blood redistributions to active muscles, perhaps, due to greater reductions in inactive muscle oxygenation, resulted in higher trend of VO_2peak_ (Yano et al. 2005). However, we must acknowledge that we can only speculate this hypothesis. Future studies should be examined to further examine potential underlying mechanisms that could explain the impact of BR supplementation on hypoxic exercise performance.

### Methodological considerations

Several limitations should be considered in the interpretation of our results. First, we must acknowledge the relatively small sample size. Based on our power analyses, we estimated that 9 subjects were necessary to achieve the appropriate statistical power for NIRS comparisons (HHb in active muscle during EX_sub_, StO_2_ in inactive muscle during EX_max_), while 10–13 would have been required for time‐to‐exhaustion and cardiorespiratory variables (*V*CO_2_ during EX_sub_ and HR_peak_ during EX_max_) (G*Power 3.1). However, 8 of the 9 subjects showed an improvement in exercise tolerance (Fig. 2), and the statistical power was high enough for NIRS signals. Although future studies are needed, it is unlikely that such additional data would strongly affect our conclusion. Second, we cannot completely rule out the contamination of the NIRS signal by skin perfusion (Davis et al. 2006). To correct for this, we measured SkBF in the active muscles during exercise. As a result, no differences were observed in SkBF between PL and BR conditions. Moreover, there were no differences in MAP during exercise between the conditions. These results indicate that the effects of cutaneous circulation on NIRS signals were similar between the conditions. Third, we did not perform ischemia calibration to normalize the NIRS signals to evaluate maximal physiological changes; however, we found no differences in arbitrary unit values at baseline between PL and BR, moreover, our analysis has been conducted in our recent study (Horiuchi et al. 2016). In our preliminary test, arterial occlusion above 200 mmHg for 10–15 min caused strenuous pain and nausea for subset of subjects, possibly, due to after exhaustion under hypoxic exercise. Thus, we were not able to perform arterial occlusion method for ethical problems. In addition, a previous study demonstrated that muscle oxygenation and deoxygenation status at vastus lateralis during leg cycling showed similar values in comparison between modified beer lambert and time resolved spectroscopy method that can allow to assess absolute values (Saitoh et al. 2010).

Similarly, NIRS can represent only the balance between O_2_ delivery and utilization, not blood flow, and therefore it could not explain the actual blood flow redistribution between active and inactive skeletal muscles. While this may be one of the limitations to interpreting our results, NIRS also has several important advantages. For example, this technique provides continuous measurement of oxygen availability at the level of microcirculation, the part of the vascular tree most accessible to metabolic products of contraction (Mancini et al. 1994). In addition, optodes placement is sufficiently stable to acquire measurements even during high intensity dynamic exercise with large muscle groups. Finally, as we did not assess the effect of nitrate supplementation on normoxia trial, it remains unclear how nitrate supplementation affect muscle oxygenation status under normoixa. However, the main aim of this study is to investigate effects of dietary nitrate supplementation on exercise performance and muscle oxygenation status between active and inactive muscles under hypoxic exercise, and our results clearly demonstrated significant differences in these outcomes. Nonetheless, we must acknowledge that our results is limited under hypoxic exercise, and that future studies are required.

In conclusion, our results demonstrated that 4 days of dietary nitrate supplementation improved exercise tolerance during hypoxic exercise. Additionally, nitrate supplementation suppressed the increases of HHb in active muscles at moderate work rate. Meanwhile, accentuated reductions in HbO_2_ and StO_2_ were observed in inactive muscles at higher work rates during EX_max_. Collectively, these findings indicate that short‐term dietary nitrate supplementation improved hypoxic exercise tolerance, perhaps, due to suppressed increases in HHb in active muscles at moderate work rates. Moreover, nitrate supplementation caused greater reductions in oxygenation in inactive muscle at higher work rates during hypoxic exercise.

## Conflict of Interest

None declared.

## Data Accessibility
